# Terpolymer resin containing bioinspired borneol and controlled release of camphor: Synthesis and antifouling coating application

**DOI:** 10.1038/s41598-020-67073-8

**Published:** 2020-06-25

**Authors:** Jiankun Hu, Baoku Sun, Haichun Zhang, Ading Lu, Huiqiu Zhang, Hailong Zhang

**Affiliations:** 1 Zhejiang Ocean Development Research Institute, No. 10, Tiyu Road, Lincheng, Zhoushan 316021 China; 2 Institute of Innovation & Application, Zhejiang Ocean University, No. 1 Haida South Rd, Lincheng, Changzhi Island, Zhoushan 316022 China

**Keywords:** Pollution remediation, Polymers

## Abstract

Marine biofouling can cause a biocorrosion, resulting in degradation and failure of materials and structures. In order to prevent sea creatures from attaching to the surface, in this work, a new environmentally friendly antifouling coating by incorporating antibacterial polymers and natural antifouling agents has been designed and synthesized. Surface chemical composition and changes in surface hydrophobicity were studied by FTIR spectroscopy and contact angle measurements, respectively. Measurements of mass loss of antifouling resin were also carried out and the release rate of camphor from antifouling coating was tested by using UPLC. It had been found that the changes in the content of triisopropylsilylacrylate (TIPSA) (from 4% to 12%) and isobornyl methacrylate (IBOMA) (from 50% to 16.7%) did not significantly affect the release of camphor. The content of IBOMA decreased from 50% to 16.7%, the antifouling performance of the resin system appeared slightly reduced. In addition, rosin could help regulate the release rate of the resin system to desorb camphor slowly in water in a controlled manner. Furthermore, the antifouling capability of as-prepared samples was evaluated via algae suppression experiments and marine field tests. This study highlighted the environmentally friendly antifouling coating as a potential candidate and efficient strategy to prohibit biofouling in seawater.

## Introduction

Submerged hull surface is highly susceptible to biofouling. Marine fouling organisms attach to the hull surface, grow and propagate, which will lead to the aggravation of surface corrosion, the exacerbation of surface friction resistance, the increase of fuel consumption, and a great number of economic loss^1^. In order to prevent marine organisms from attaching and adhering, a variety of methods have been adopted, such as physical method, chemical method, net changing method, new antifouling nets^2,3^. Among those technologies, antifouling coating is the most convenient, effective and economical method. Due to its low cost and easy implementation, antifouling coating has always been the direction of efforts to solve the problem of antifouling^4^. In recent decades, a variety of functional polymer brushes and coatings have been developed to prohibit the attachment of marine organisms^5^. However, traditional antifouling coatings rely on the release of antifouling agents to control biological adherence, which seems to show a promising antifouling effect^6^. Unfortunately, these antifouling agents are toxic molecules, because of the toxic nature of certain biocides such as tributyltin (TBT) and copper, they can cause marine pollution and bring about serious harm to the ecological environment^7^. Since 2008, the International Marine Organization (IMO) has constantly urged and continuously strengthened legislation to restrict the use of toxic antifouling agents^8^. Therefore, it is urgent to develop environment-friendly, non-toxic or low toxic antifouling compounds^9,10^. In the past decade, environmentally friendly antifouling coatings based on self-polishing copolymers and natural antifoulant have drawn substantial attention as a promising strategy and the most effective means of sequestering biocorrosion and biofouling^11,12^.

Natural compounds separated from marine microbes, algae, aquatic plants, marine invertebrates, and land and other sources are a promising source of antifouling agents^13^. Compared with synthetic antifouling agents, they have the advantages of compatibility with biological systems, and are more specific to bacteria, algae and mollusks and other attached organisms than heavy metals^14,15^. However, despite the isolation of many potential antifouling compounds from marine natural products, their application as effective antifouling biocides has been progressed slowly^16^. Among these natural antifouling agents, 2,5,6-tribromo-methylgramine deserves consideration because of its simple structure and high antifouling capability^17^. It has been reported that the coatings with poly(m-aminophenol) (PmAP) have improved antifouling performance which can effectively inhibit the formation of biofilm^18^. There are still many challenges to be overcome in terms of cost, large-scale production, biosafety and pollution control mechanisms. In addition, the introduction of antifouling compounds into synthetic polymers and the release of natural antifouling agents from coatings are of great importance for the reliability and longevity of metals in service. And research on the synthesis and property of its analogues has attracted increasing attention^19^.

Camphor is a terpene natural organic compound, which can be extracted from the trunk of *Cinnamomum camphora* and can also be synthesized in large quantities^20^. At room temperature, it appears white or transparent waxy solid. It is often used in the practice of daily life to repel insects and mosquitoes^21^. Theoretically, it is a potential compound with antifouling ability^22^. If one or more compounds or polymers can be designed, synthesized or blended to make them compatible with camphor, with stable controlled release of camphor and synergistic anti-fouling effect, it will be of great significance to the application of environmentally friendly antifouling coatings^23,24^. Borneol is a time-honored herb in traditional Chinese medicine, a crystalline cyclic alcohol that occurs in two enantiomeric forms. It can be extracted from medical herbs such as lavender, lavenrian, and chamomile^25^. It has many biological effects such as sedative, antiinflammatory, analgesic, anti-nociceptive, antithrombotic and vasorelaxant effects^26^. Natural herb-based antibacterial borneol has been reported to have an excellent broad-spectrum antibacterial capability^27^. Borneol-based polymers, isobornyl methacrylate (IBOMA), have been demonstrated a strong activity against bacterial infection and can be suitable for preparing antibacterial coating^28^.

The novel approach employed in the present study is synthesis of an environmentally friendly antifouling coating based on antibacterial polymer (such as IBOMA) and natural antifouling agent (such as camphor). The as-prepared coatings can slowly degrade and release borneol in seawater, while borneol itself has antibacterial properties, and at the same time, it provides a self-renewing surface to prevent fouler from adhering. Furthermore, antifouling camphor is also released together, providing a special antibiosis and antifouling surface with sterilized polymers to play a synergistic antifouling effect. The present study aims at the investigation of the antifouling properties of as-prepared coatings and synergistic antifouling effect of polymer hydrolysis, camphor and borneol release.

## Experimental

### Materials

Triisopropylsilylacrylate (TIPSA) was provided by Yangzhou Upkind Technologies Co., Ltd (Jiangsu, China). Isobornyl methacrylate (IBOMA), n-butyl methacrylate (BMA), camphor, azo-iso-butyronitrile (AIBN), 2,2’-azobis-(2-methylbutyronitrile) (AMBN), benzoyl peroxide (BPO), 1-dodecanethiol, camphor, xylene (Xy) were received from Xiya Regent Chemical Co. (Shandong, China). Artificial seawater (ASW) was prepared according to ASTM D1141-98 (2013)^29^. The chemicals used in this study were analytical grade and were used at the time of reception without further purification.

### Synthesis of antibacterial polymer

**Figure 1:**
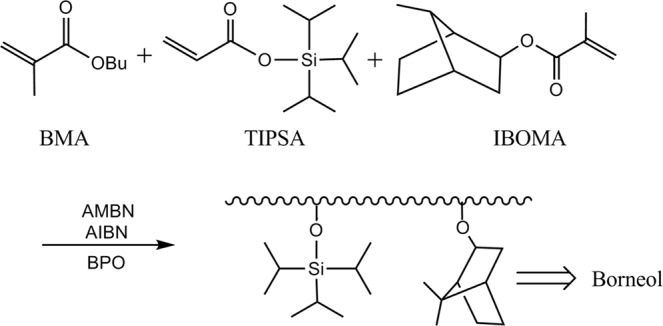
Synthesis of terpolymer resin. Synthetic routes of resin compounds, polymer (IBOMA) releases antibacterial borneol through hydrolysis.

Antibacterial polymers were synthesized by polyaddition as illustrated in Fig. 1. In a temperature-controlled reaction vessel equipped with a condenser, a thermometer, a dropping funnel and a stirrer, Xy (50 g) and AMBN (0.5 g) were added and heated to 100 °C under stirring. From the dropping funnel, a transparent mixture consisting of TIPSA, IBOMA, BMA (their proportion as illustrated in Table 1), Xy (240.0 g), dodecanethiol (3.0 g), AIBN (2.0 g) and AMBN (2.0 g) was dropped at a constant rate over a period of 6 h. After the completion of dropping, Xy (10 g) and BPO (0.5 g) were further dropped over a period of 30 min, and then the reaction vessel was heated to 110 °C and stirred for 3 h. The series of antifouling resins were synthesized and samples were named as shown in Table 1. Camphor, a natural antifouling agent, was added to each sample at a ratio of camphor to resin, 1:10, and mixed extensively. Then each resin sample was coated on a clean glass plate, 25 × 75 mm^2^. The sample 3c was prepared by adding 10% rosin in sample 3 and mixing well.

**Table 1:** Compositions of resins prepared by free radical copolymerization.

Terpolymer resins	1	2	3	4	5
TIPSA/g	12	24	36	36	36
IBOMA/g	150	150	150	100	50
BMA/g	138	126	114	164	214
Xylene/g	300	300	300	300	300

Note: on the basis of sample 3, add 10% rosin to get sample 3c.

### Swelling and degradation evaluation

A gravimetric method was employed to monitor *in-situ* the degradation and swelling of each coating, i.e., sample 1, 2, 3, 3c, 4, and 5. Each glass slide used was weighted before and after the coating to determine the initial dry weight (W_0_) of each sample. Three slides of each coating were used to get an average value and deviation. Immersed in an ASW container which was renewed every other day, each coated glass slide was weighted after a certain period of time. After removed from the ASW container, the slide was rinsed with distilled water for 5 min and then gently dried before its weight (W_t_, wet) was recorded. Then, after immersed for a period of time, these glass slides were dried at 25 °C for one day and then at 100 °C for 6 h before their weights (W_t_, dry) were recorded. This procedure was repeated on day 14, 30, 48, 56, and 64. The weight difference between W_t_, wet and W_0_ reflected the swelling of each coating. And, the dry weight difference between W_0_ and W_t_, dry showed the degradation (self-peeling) of each coating, which could be converted to the thickness change (thickness) for a given density and coating volume.

### Determination of the release rate of camphor

The release rate of camphor was determined by measuring its concentration using UPLC under a static condition^30^. Typically, a composite coating of antifouling resins was prepared on a glass panel (25 × 75 mm^2^), and then the panel was immersed in artificial seawater (ASW). After a given period of time, the panel was transferred from the holding tank to an individual measuring container with 100 mL of fresh seawater. After 24 h immersion, 10 mL of seawater was taken from the individual measuring container and extracted three times using 10 mL of trichloromethane. After drying under nitrogen gas, the extract was resuspended in 100 μL of methanol and then subjected to HPLC analysis. The camphor peaks were determined from its retention time, and its amount was calculated from the established standard curves using peak areas plotted against known quantities of standards. The recovery efficiency for camphor analysis was 85.6%.

### Characterization

The molecular weights for all terpolymer resins were determined by Gel Permeation Chromatography (GPC, Malvern, ViscoTEK TDA, Houston, England) measurements in THF. The calibration was based on polycal PMMA standards (Both Mn = 51092, PDI = 1.038 and Mn = 95934, PDI = 1.838 were used to the calibration curve).

IR spectrum was recorded on a Fourier transform infrared (FTIR) spectrometer (TENSOR II, BRUKER, USA). Analyses were performed in the transmission mode in the range 4000-500 cm^−1^ at room temperature with a resolution of 2 cm^−1^ and accumulation of 16 scans. Surface roughness was assessed by using an ultra-depth-of-field electron microscopy (KEYENCE VHX-6000, Keyence Corporation, Osaka, Japan).

Contact angle measurements were performed by using a contact angle measuring instrument (SINDIN SDC-200S, Dongguan Shengding Precision Instrument Co. LTD., Dongguan, China) with an automatic dispenser. The contact angle of each sample was measured after it was taken out from the sea water, tilted and placed for a period of time (e.g., 30 minutes here), and dried naturally on the surface. A droplet (about 25 ml) of Mili-Q water was placed on the surface of each sample; then the liquid was withdrawn immediately. The average of right and left contact angles (denoted “contact angle” in this work) was measured and calculated from 3 pictures taken each second, using *Young- Laplace* fitting method. Contact angles of 4 different spots on the surface were measured and the final value of contact angle was the average of contact angles at these 4 spots.

The release rate of camphor was determined by measuring its concentration using ultra performance liquid chromatography (UPLC), a phase system (Waters UPLC H-Class, Waters Technologies Shanghai Limited, Shanghai, China) with an Acquity UPLC Behc C18 column connected to a photodiode array detector at 289 nm.

Experiments on inhibiting algae attachment were carried out and fluorescence photographs were taken by an inverted fluorescence microscope (Leica DMi8, Leica Mikrosysteme Vertrieb GmbH, Wetzlar, Germany).

### Experimental method of inhibition of Chlorella adhesion

Shake the algae solution of *Chlorella* in exponential growth period and pour it into a sterilized algae culture container. Dip as-prepared coating samples (cover the back with aseptic tape) into the algae culture container, tilt to the side and place it downward. Seal the culture container with an aseptic sealing film with air vent on the surface. Carefully move the container into an illuminating incubator. In the incubator, the temperature was controlled at 23 °C, the ratio of light-to-dark was set to 12:12, and the light intensity was 3960 lx. After 2 days of stationary culture, take out the sample and place it on its back. After the sample surfaces are dried, the attachment of *Chlorella* on the coating surfaces is observed by the inverted fluorescence microscope. Because *Chlorellas* adhere on the coating surface randomly, fluorescence images are taken by random selection of 10 points on the coating surface. The attachment area of algae is calculated by image processing software and the data of 10 points are averaged. The attachment rate (attachment area divided by total area x 100%) has been used as the evaluation index of antifouling performance of the sample.

### Marine field tests

The antifouling capacity test was carried out in accordance with the national experimental standard for marine antifouling coatings at shallow sea^31^. Each resin sample was applied on a steel substrate, 300 × 250 × 3 mm^3^. The bottom layer was epoxy zinc rich primer, and the top/finish layer was as-prepared antifouling coating for testing. Three sets of parallel samples were prepared for each coating to test their antifouling performance at different depths of the ocean. After preparation of each coating, the four corners of samples were fixed on hangers with stainless steel bolts. After the installation was completed, the hangers were placed on an experimental floating raft in the testing area, which was located in the offshore of Louman’s sheltered bay, Zhoushan. It was a subtropical sea, and the velocity of sea water was lower than 5 m/s, the azimuth: 30°01’ N, 122°06’ E, the test depth: 0.3 − 2.0 m, the main fouling organisms attached to the board usually were barnacle.

## Results and Discussion

### Analysis of FTIR

**Figure 2:**
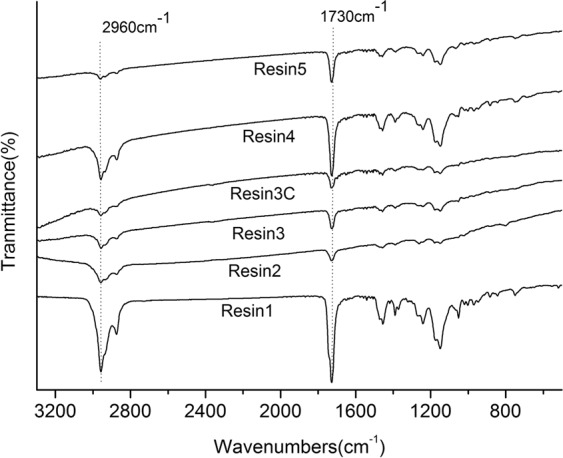
The chemical structure and composition of samples. FTIR spectra of the resin samples.

The chemical structure and composition of as-prepared resin samples was studied using FTIR absorption spectroscopy. Here resins were used without camphor in order to facilitate the comparison of their chemical components of samples. In Fig. 2 for anti-fouling resins characteristic bands were observed at 2960, 1730 and 1150 cm^−1^. The stretching vibration absorption peaks of C = C double bond (1650 cm^−1^) did not appear in the spectra, indicating that the copolymer of binary acrylic resin had been polymerized. 2963, 2907 and 2873 cm^−1^ were the stretching vibration absorption peaks of saturated C-H, 1730 cm^−1^ was the stretching vibration absorption peak of C = O, 1450 and 1390 cm^−1^ were the in-plane bending vibration absorption peaks of C-H. 1240 cm^−1^ might be the out-of-plane bending vibration absorption peak of C-H, and medium and wide doublet peaks, 1180 and 1150 cm^−1^, were the stretching vibration absorption peaks of C-O-C. Table 2 illustrated the GPC traces of the molecular weights of all prepared samples. The average molecular weight and the dispersity of all samples were only little difference. The average molecular weight of samples 1, 2 and 5 was slightly larger than that of samples 3 and 4.

**Table 2:** GPC of anti-fouling resins.

Sample	*M*_w_ (GPC)	*M*_n_ (GPC)	PDI = *M*_w_/*M*_n_
**1**	34790	18258	1.905
**2**	34332	18893	1.817
**3**	23802	14512	1.640
**4**	22112	14072	1.571
**5**	31999	18941	1.689

### Quality loss test

Measurements of the mass loss of antifouling resins were carried out and the release rate of camphor from antifouling coating was tested by using UPLC. The weight loss rate of resin and the biocide release rate of resin containing camphor were the two key factors which determined the duration and antifouling performance of an antifouling system.

**Figure 3:**
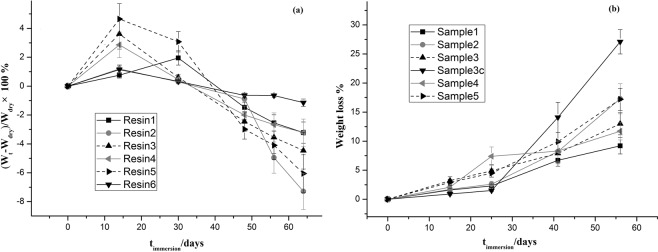
The mass loss of resins. (**a**) The swelling rate of resin; (**b**) The weight loss rate of antifouling coatings.

As shown in Fig. 3(a) that in the early stage of immersion, the mass of each sample increased to a certain extent, among which sample 3, 4 and 5 increased the most. The possible reason was that samples 3, 4, and 5 contained relatively high content of TIPSA, and the water absorbability was relatively stronger. After more than 60 days of immersion, all the samples appeared to have a certain degree of weight loss; however the results showed that the weight loss rate was stable within an error range, indicating that the change of TIPSA, IBOMA and BMA content had no significant effect on the weight loss rate of the resin. Especially compared with sample 1, the system had no obvious hydrophilic group, and sample 1 has the best water resistance in the group. Therefore, the weight loss rate of sample 1 was the lowest.

Fig. 3(b) illustrated the time dependence of the weight loss of as-prepared coatings on glass panels immersed in ASW. When the resin and the camphor were mixed, the changes were slightly different. In the first stage, the weight loss of each sample was basically the same. The weight loss of sample 3c with 10% rosin was slightly lower than that of other samples, but the difference was not significant. In the second stage, the weight loss of sample 4 increased rapidly. The weight loss of sample 3 was lower to that of sample 4, while the weight loss rate of other samples did not change significantly. It might be that the molecular weight of sample 3 and 4 was relatively smaller, and the content of TIPSA was higher. The smaller the molecular weight and the easier the hydrolysis of TIPSA might result in the difference of the local hydrolysis of the resin. In particular, sample 4 had the smallest molecular weight and dispersion, which might lead to the rapid increase of weight loss rate.

In the second stage, compared with sample 3c, basically it continued the trend of the first stage, and the weight loss was still the lowest, which indicated that the resin might have a certain delay effect on the weight loss of coating system. The possible reason was that in the presence of seawater, resin had a better hydrophilicity than camphor. The TIPSA segment might preferentially migrate to the coating surface, reducing the exposure of camphor to seawater, thus delaying the release rate of internal camphor and reducing the weight loss rate.

In the third stage, the release rate of sample 3c increased rapidly, but the variation tendency of other samples was not obvious. Sample 4 returned to the normal group of weight loss. This indicated that the proportion of TIPSA in the whole system was limited, which was not enough to change the weight loss effect of the whole system. In the fourth stage, sample 3c continued its rapid release trend in the previous stage, while other samples also had some increase, but their changes were inferior to sample 3c. This suggested that rosin might help sample 3c release camphor in the third and fourth stages. The rosin might play a certain proactive role in the release of camphor in the middle and late stage. The possible reason was that with the prolongation of immersion time, the rosin gradually degraded and released into the seawater, forming a hollow channel in the resin system, which in turn was conducive to the release of the internal camphor, improving the release rate of camphor in the later stage, thus further increasing its weight loss. This also followed the principle of the release antifouling paint that had been applied up to now^32^.

### Analysis of contact angle and surface roughness

**Figure 4:**
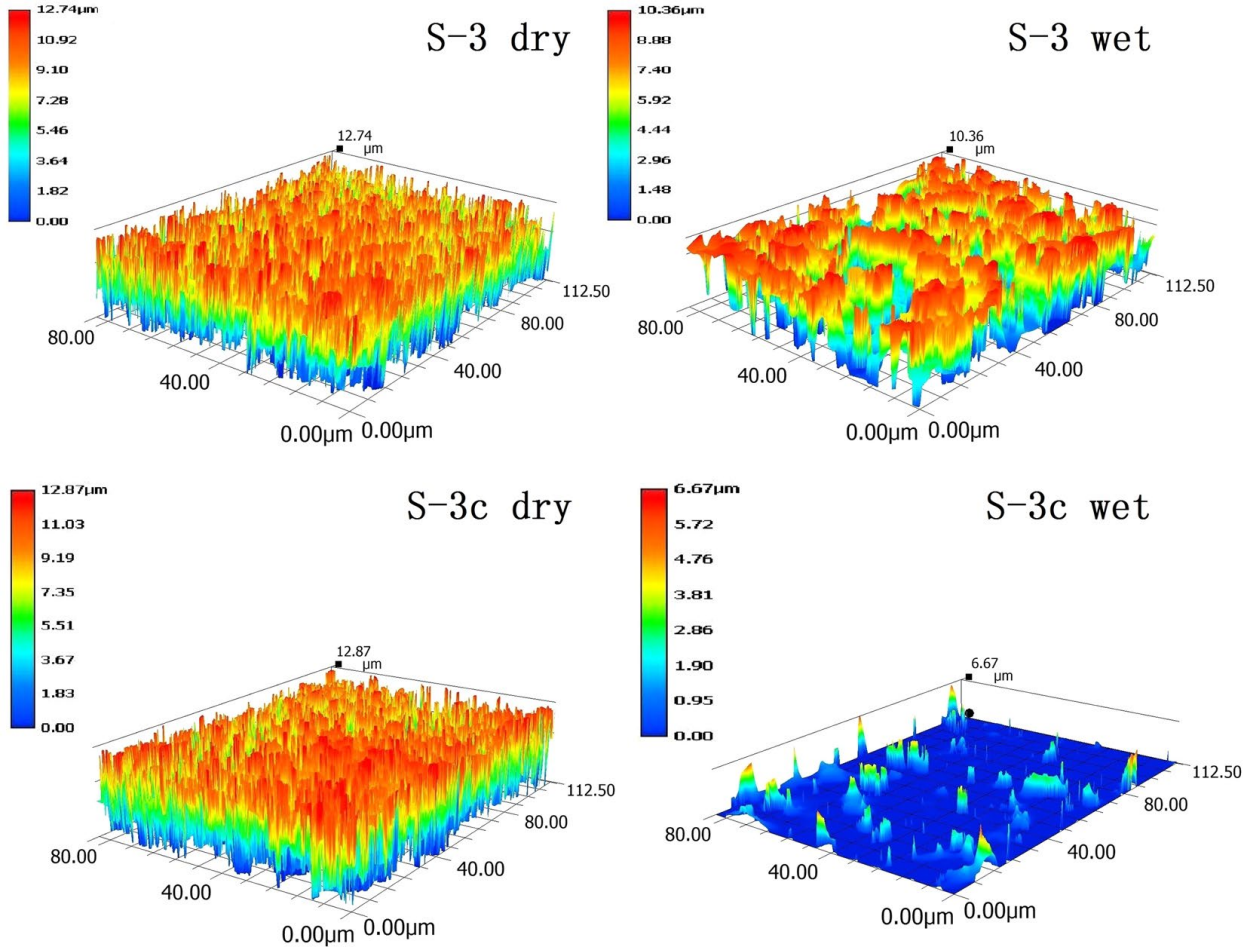
Surface roughness measurements. The images, sample 3 (S-3, above), and 3c (S-3c, bottom), of surface roughness of anti-fouling resins taken from an ultra-depth-of-field electron microscopy.

In order to evaluate the role of surface characteristics of the coatings, their surface properties before and after immersion were analyzed by measuring contact angle and surface roughness. As showed in Table 3, after immersion in ASW, the contact angle of all samples decreased significantly, e.g., the contact angle of sample 3c decreased from 94 degree to 67 degree. As indicated in Table 4, the roughness of all samples also appeared a similar trend. After immersion in ASW, the maximum value of surface roughness of sample 3c from dry surface, 12.87 μm, reduced to 6.67 μm for its wet surface. These changes could also be visualized for sample 3 and 3c as examples as illustrated in Fig. 4. This might be due to the hydrolysis of acrylic silicone resin and the increase of carboxylic acid radical content, resulting in the increase of hydrophilic characters. The contact angle of sample 3c (with extra 10% rosin) decreased most. These might suggested that the auxiliary resin itself was hydrophilic, which further enhanced the hydrophilicity of the coatings. These hydrophilic and slippery surfaces would further reduce the chance of the initial attachment of foulers^33^.

**Table 3:** Surface contact angles of anti-fouling resins.

Sample	1	2	3	4	5	3c
dry (°)	94	93	93	93	91	94
wet (°)	78	83	79	77	82	67

**Table 4:** Surface roughness of anti-fouling resins.

Sample	1	2	3	4	5	3c
**dry (μm)**	12.83	12.38	12.74	12.85	12.72	12.87
**wet (μm)**	9.34	7.75	10.36	7.61	8.13	6.67

Note: ‘dry’ represents the surface state of coatings after fully cured; ‘wet’ represents the surface state of coatings after immersion in water for 7 days and after surface water has been removed naturally.

### Controlled release of camphor

In order to optimize the antifouling performance of an antibiofouling system, it is necessary to study the controlled-release performance of antifoulant camphor in detail. Since the resin is not completely hydrolyzed, the release rate of antifouling agents is slower than that of more commonly used self-polishing resins^34,35^. Therefore, it is worth noting that it is inappropriate to use 24 hours to detect/test the release rate of the system in some other literatures.

**Figure 5:**
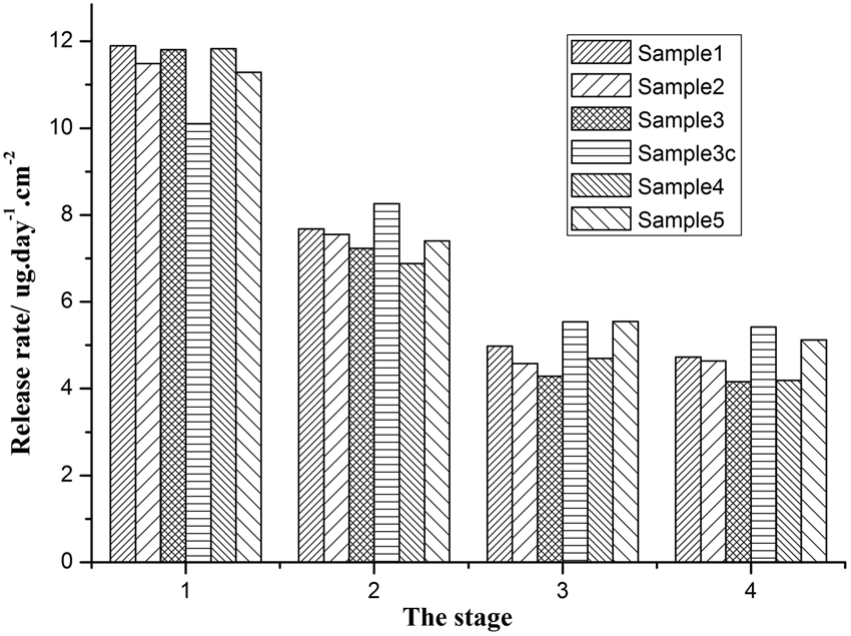
Controlled release of camphor. Release rate of camphor in resins at different stages.

In order to resolve the problems of slow release rate and a lot of error, here we use a staged method to calculate the average value of multi-day cumulative release. In our experimental system, the total process of release rate can be divided into four stages, the first stage is 1–25 days, and the other stages are 26–41, 42–56 and 57–72 days, respectively. It can be seen from Fig. 5 that the average release rate of the first stage is the highest for all samples, and the average release rate then decreases gradually with time. In the third and fourth stages, the release rate gradually stabilized. Therefore, the first stage can be regarded as the period of high speed release, the second stage is the period of transition release, the third and the fourth stages are the periods of stationary release, which means that the release of antifouling agents in the resin system tends to be stable.

During the first stage, the high speed release, as shown in Fig. 5 the release rates of sample 1, 2, 3, 4 and 5 are all around 11 μg/day·cm^2^, the release rate of sample 3c containing an extra 10% rosin is obviously lower than that of other samples. This illustrates that the rosin has a certain delay effect on the release of camphor in the period of high speed release stage during the first stage. The possible reason is that rosin has better hydrophilicity than camphor in the seawater environment, and preferentially it migrates to the top surface of the coating, so as to reduce the chance of camphor contacting with seawater and delay the release rate of internal camphor.

During the second stage, the transition period, the release rates of samples 1, 2, 3, 4 and 5 are all around 7. Compared with other samples, the release rate of sample 3c is higher. In the third and fourth stages, during the period of stationary release, the release rate of each sample tends to be stable. However, the release rate of 3c is still slightly higher than that of other samples, although this advantage is not obvious. This shows that in the middle and later stages the release of the resin has a certain promoting effect on the release of camphor. The possible reasons are that with the prolongation of the soaking time, the resin is gradually released into the seawater, forming a hollow channel in the resin system, which is beneficial to the diffusion and release of the internal camphor, so as to improve the release rate of camphor at the late stage^3^. The behaviour and properties of as-prepared coatings are in accordance well with the principle of releasable antifouling coatings^32^. At the same time, Fig. 5 also shows that the changes in the content of silicon acrylate monomers (TIPSA), which increase from 4% to 12%, and IBOMA, which decreases from 50% to 16%, do not significantly affect the release amount of camphor. In addition, the antifouling performance of all samples has been further evaluated via algae suppression and marine field tests.

### Analysis of algae suppression data

**Figure 6:**
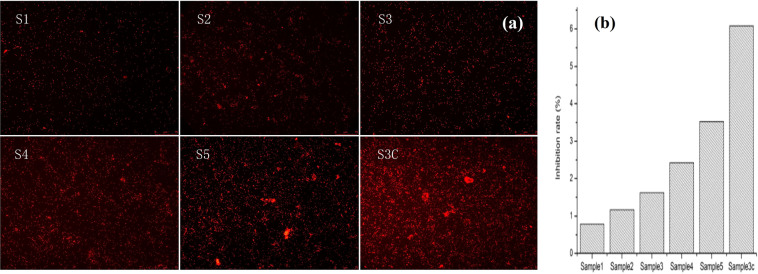
Algae inhibition experiments. (**a**) Fluorescence images of as prepared coating samples after inhibition test of algae adhesion; (**b**) Inhibition rate of algae calculated via the attachment areas of algae.

As can be seen from Fig. 6, the antifouling test of algae cell attachment illustrates that the coatings of environmental friendly biocides have good and encouraging anti algal adhesion performance. The algae attachment intensities of sample 1, 2, 3, 4, 5 and 3c are 0.781%, 1.168%, 1.622%, 2.427%, 3.526%, 6.083% respectively, as shown in Fig. 6b. With the increase of silicon content in acrylic resin, the algae adhesion intensities of the sample increases gradually, and the algae inhibition ability of the coating decreases with the increase of silicon content in acrylic resin. Silicon surface is inert to microbial attack but fungal or algal growth occurs especially when extra carbon sources are available as nutrients, which is the case for acrylic resin^36,37^. On the other hand, with the decrease of isobornyl methacrylate monomer content, the inhibition ability of algae decreases, as indicated in Table 1, sample 1, 2 and 3 with 150 g of IBOMA, and only 100 and 50 g of IBOMA in sample 4 and 5, respectively. Because IBOMA can release borneol via hydrolysis, borneol has a remarkable inhibitory effect on bacterial attachment and growth^26^. And therefore borneol based polymers can be utilized for fabricating biocompatible antibacterial coating interface^28^.

### Marine field tests

**Figure 7:**
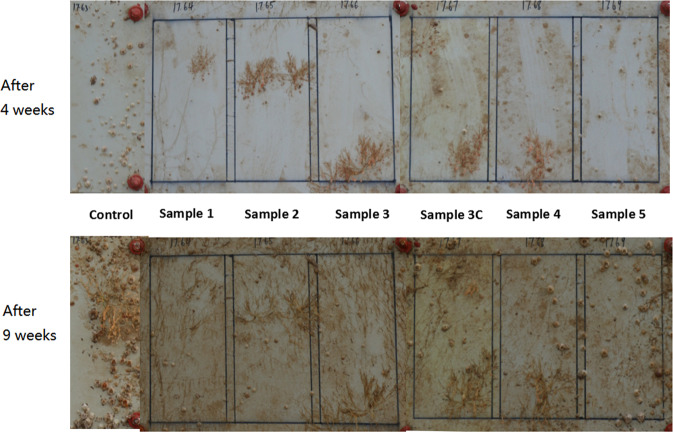
Marine field tests. Morphologies of the antifouling performance of coating samples at different stages, (above, 4 weeks; bottom, 9 weeks).

In order to further assess the antifouling properties of resin samples, the short-term antifouling performance of all as-prepared coating samples was evaluated by marine field test. In Fig. 7, the typical images of panels coated with as-prepared coating samples were illustrated. When the content of IBOMA was the same for sample 1–3, with the increase of TIPSA content, the antifouling performance of the coating was not obviously improved. The possible reason was that both IBOMA and TIPSA were small functional monomers with some antifouling effects. The small change of content might not be enough to influence the antifouling performance. When the content of TIPSA was the same, the content of IBOMA decreased from 50% to 16.7%, indicating that the amount of released borneol could decrease. Therefore, the antifouling performance of the resin system could decrease slightly. This was also consistent with the above algae suppression data. The decrease of IBOMA content was accompanied by the decrease of antifouling performance of antifouling coatings, which suggested that IBOMA had better antifouling performance than TIPSA. In addition, resin could help regulate the release rate of the resin system by reducing the release rate of antifouling agents in the early stage and accelerating the release rate of antifouling agents in the later stage, which was the key process to prevent the occurrence of biofouling in the long run^31^. From the actual marine testing, the change of release rate could not significantly alter the antifouling performance of the resin system. After 4 weeks, there almost none of barnacle appeared on the surfaces of sample 1–3, there were barely a few barnacles on the surfaces of sample 4–6. However, there were many more barnacles attached on the surface of control samples. Here marine field tests did not seem to follow the same trend with those from analysis of algae suppression. In addition, all the samples seemed to have yellow brown adherents’ appearance on the surfaces, which were likely to be silts or tiny grain aggregates in the sea water. Generally, the seawater near Zhoushan emerged muddy or turbid water with sediments. How to overcome this problem might require further study.

**Figure 8:**
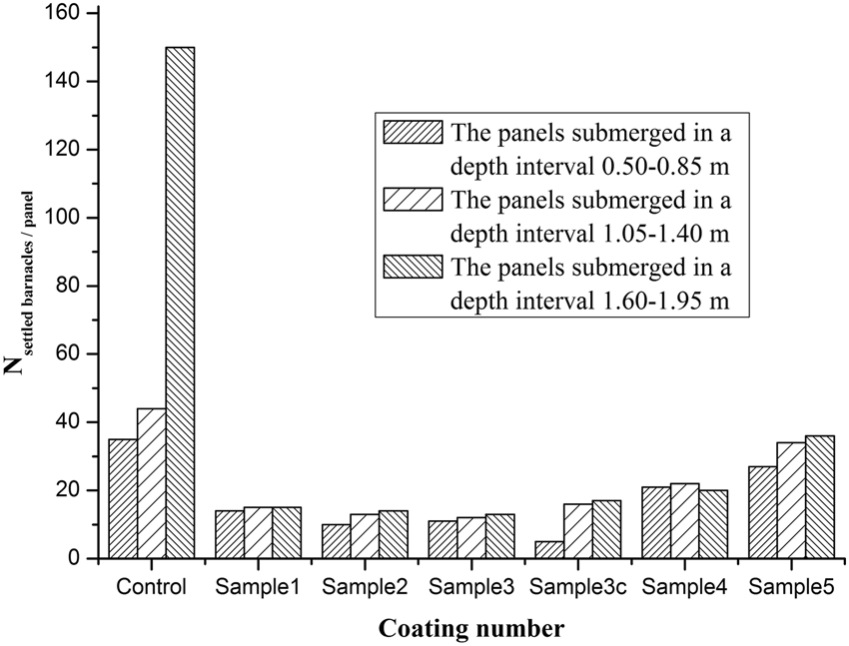
Evaluation of antifouling performance. The number of barnacles attached to antifouling boards.

Fig. 8 illustrated the number of barnacles attached to antifouling plates. In Fig. 8, the number of barnacles on the control samples appeared to be the highest after 9 weeks in the marine field, especially for the control panel submerged in a depth interval 1.4–1.75 m. As discussed above, the release rate of borneol increased as the content of IBOMA increased, so that the antifouling performance of resin samples could be improved. Because of their hydrolysis of ester bonds, the antifouling performance is mainly dependent on the self-renewal of as-prepared coatings and the synergistic effect of the antimicrobial borneol released from the coatings and the desorption of camphor, a nature antifoulant.

## Conclusion

A new polymer antifouling coating has been successfully synthesized by using environmentally friendly terpolymer resins and natural antifouling agent camphor. It has been demonstrated that our antifouling polymer can be released slowly in a controlled manner. Due to the hydrolysis of acrylic silicone resin and the increase of carboxylic acid radical content after immersion, the hydrophobicity of the coating surface rapidly changes to hydrophilicity. The application performance of this antifouling system also has been tested. The changes of TIPSA content have no significant effect on the release rate and antifouling performance of the resin system. Although the change of IBOMA content does not significantly affect the release rate of camphor, the decrease of IBOMA content corresponds to the antifouling performance of the resin system. Rosin may help regulate the release rate of resin system by reducing the release rate of camphor in the early stage and accelerating the release rate of camphor in the later stage. The results of marine field tests highlight the potential application of the polymer antifouling system synthesized by incorporating antibacterial polymer and natural antifouling agents to prevent fouling in the application of antifouling coatings. The results need to be further evaluated via marine field tests for longer duration.
